# Regulatory miRNAs in Colorectal Carcinogenesis and Metastasis

**DOI:** 10.3390/ijms18040890

**Published:** 2017-04-22

**Authors:** Yongchen Guo, Yonghua Bao, Wancai Yang

**Affiliations:** 1 Department of Pathology and Institute of Precision Medicine, Jining Medical University, 16 Hehua Road, Jining 272067, China; guoyongchen2005@126.com (Y.G.); baoyonghua2005@126.com (Y.B.); 2 Department of Pathology, University of Illinois at Chicago, Chicago, IL 60612, USA

**Keywords:** colorectal cancer, miRNA, epithelial-mesenchymal transition (EMT), cancer stem cells, metastasis

## Abstract

Colorectal cancer is one of the most common malignancies and is the second-leading cause of cancer-related death world-wide, which is linked to genetic mutations, epigenetic alterations, and oncogenic signaling activation. MicroRNAs, one of the categories of epigenetics, have been demonstrated significant roles in carcinogenesis and progression through regulating of oncogenic signaling pathways, stem cells, epithelial-mesenchymal transition, and metastasis. This review summarizes the roles of microRNAs in the regulating of Wnt, Ras, TGF-β, and inflammatory signaling pathways, stemness, and epithelial-mesenchymal transition, for carcinogenesis and metastasis in colorectal cancer. Improving our understanding of the mechanisms of regulatory interactions of microRNAs with signaling pathways in colorectal cancer formation and progression will aid in determining the genes responsible for colorectal cancer initiation, progression, metastasis, and recurrence and, finally, in developing personalized approaches for cancer prevention and therapy.

## 1. Introduction

Colorectal cancer (CRC) is one of the most common malignancies and is the second-leading cause of cancer-related death worldwide [1]. Although several decades of effort have been made, the underlying mechanisms are still largely unknown. However, more and more evidence has demonstrated that colorectal carcinogenesis is linked to the activation of oncogenic signaling pathways and inactivation of tumor-suppressive signaling, resulting mostly from genetic mutations and epigenetic alterations, the latter including DNA methylation, histone acetylation, and non-coding RNAs (e.g., microRNAs, long non-coding RNAs, etc.). Among them, microRNAs (miRNAs) have shown critical biological functions according to the increasing evidence from clinical epidemiology and experimental studies [2,3,4,5,6,7]. Interestingly, these miRNAs are differentially expressed in colorectal cancer tissues, serum and plasma, and body fluids and, therefore, some miRNAs have been potentially used as biomarkers for diagnosis and therapeutic targets, exhibiting clinical importance [8,9,10,11,12]. Moreover, molecular mechanistic studies have revealed that these miRNAs participate in carcinogenesis and progression. In colorectal cancers, miRNAs have shown involvement in, or directly regulating, oncogenic signaling pathways, such as Wnt, Ras, TGF-β, and NF-κB/AKT/STAT3 signaling pathways [2,3,9,10]. In addition, these miRNAs are also involved in the regulating of the stemness of cancer stem cells, epithelial-mesenchymal transition (EMT), and metastasis [13,14,15]. Based on biological functions and regulatory interaction, we named these groups of miRNAs as regulatory miRNAs. Herein, we reviewed the regulatory miRNAs in the regulating of oncogenic signaling pathways in colorectal cancer formation and metastasis.

## 2. Clinical Significance of miRNAs in Colorectal Cancer

The studies from animal models and clinical epidemiology have shown differential expression of miRNAs in CRC or even pre-cancerous lesions. Using a colorectal cancer mouse model Muc2 knockout mice and miRNA array analysis on colonic epithelial cells, we have found that miRNAs were differentially expressed in mouse colonic epithelial cells [16], including 20 downregulated and 71 upregulated miRNAs. To confirm the accuracy, we chose the 15 most-changed miRNAs (six upregulated and nine downregulated) for validation by quantitative RT-PCR in mouse intestinal epithelial cells and, further, the mostly-changed miRNAs were validated in human colonoscopy biopsies of colitis and colorectal cancers. Bioinformatic analysis and functional studies have shown that these miRNAs mostly target the genes associated with cytokines and chemokines that are linked to inflammatory pathways and colitis-associatedcolorectal cancer [16]. Increasing studies have demonstrated the differential levels of miRNAs in human colorectal cancer tissues and serum, some are downregulated, exhibiting tumor suppressive functions, and some are upregulated, exhibiting oncogenic functions. To date, about 50 miRNAs have been found up- or downregulated in CRC cells as compared to non-tumor normal cell [12]. Some of miRNAs, such as miR-106, miR-31, miR-21, miR-25, miR-20a, miR-93, miR-183, and miR-203, are upregulated in CRC, but miR-1, miR-126, miR-30a, miR-143, miR-145, miR-191, and miR-192 are downregulated in CRC, whereas some miRNAs are also upregulated in one report and downregulated in another report in CRC, showing controversial functions. For example, miR-27a was found downregulated and showed tumor-suppressive functions in CRC, targeting Stat3 and Smad2 [16], and in other cancers [17,18] it was also found upregulated and showed oncogenic functions in CRC [19]. Moreover, cancer cells release miRNA into peripheral blood [20]. Therefore, these miRNAs could be detected from serum and plasma, andcould be used as biomarkers for diagnosis. Interestingly, the circulating miRNAs in blood have been recently found to be covered into a complexes, known as exosomes, which are protected from RNase degradation and become more stable [21].As well as detection in peripheral blood, CRC-specific miRNA could also be detected in stool. The study from Link et al. has shown that higher levels of miR-21 and miR-106a were detected in the feces of CRC and colorectal adenoma patients compared with healthy controls [22].In addition, the plasma levels of miRNAs could be used as biomarkers monitoring cancer progression or treatment. For instance, plasma levels of miR-17-3p and miR-92a were found to be reduced after surgical removal of colon cancers [22].

Therapeutic roles of miRNAs have been proven in in vitro and in vivoexperiments. As addressed above, miRNAs are aberrantly expressed in colorectal cancer tissues and cancer cell lines. Therefore, tumor suppressive miRNAs, or their mimics, can beused as novel agents for targeted therapy; in contrast, the oncogenic miRNAs can be used as targets for personalized therapy, or correcting of the aberrant expression of miRNAs by either blocking or restoring miRNA levels and functions as therapeutic strategiesfor CRC treatment. Several lines of evidence have shown that anti-cancer miRNA mimics could inhibit CRC cancer cell proliferation and migration, induce cancer cell apoptosis in vitro, and inhibit cancer cell growth in nude mice (e.g., miRNA-27a showed anti-cancer functions in colorectal cancer cells, and miR-27a mimics could inhibit CRC cell proliferation and tumor growth in nude mice [23]); restoration of miR-195 in CRC cell lines reduced cell viability, promoted cell apoptosis, and suppressed tumorigenesis [24]; and miR-342 could induce colon cancer cell cycle arrest at G0/G1 phase [25].Recent studies have also shown that some miRNAs (e.g., miR-137, miR-139-5p, miR-143, miR-409-3p, miR-494, etc.) enhanced CRC chemo-sensitivity, but some miRNAs (e.g., miR-587, miR-133a, miR-492, miR-192, miR-215, etc.) are associated with chemo-resistance in colorectal cancers [26].Furthermore, studies have also demonstrated that several miRNAs (e.g., miR-21, miR-200, miR-215, miR-143) have shown prognostic potential in colorectal cancers [27,28]. For example, miR-143 expression has been identified as an independent predictor of patient survival. Colorectal cancer patients with low levels of miR-143 expression have a significantly higher risk of having shorter cancer-specific survival and progression-free survival [29]. Taken above, miRNAs have critical clinical significance on colorectal cancer diagnosis, targeted therapy, and outcome prediction.

## 3. Regulatory miRNAs in Oncogenic Signaling Pathways

**Figure 1:**
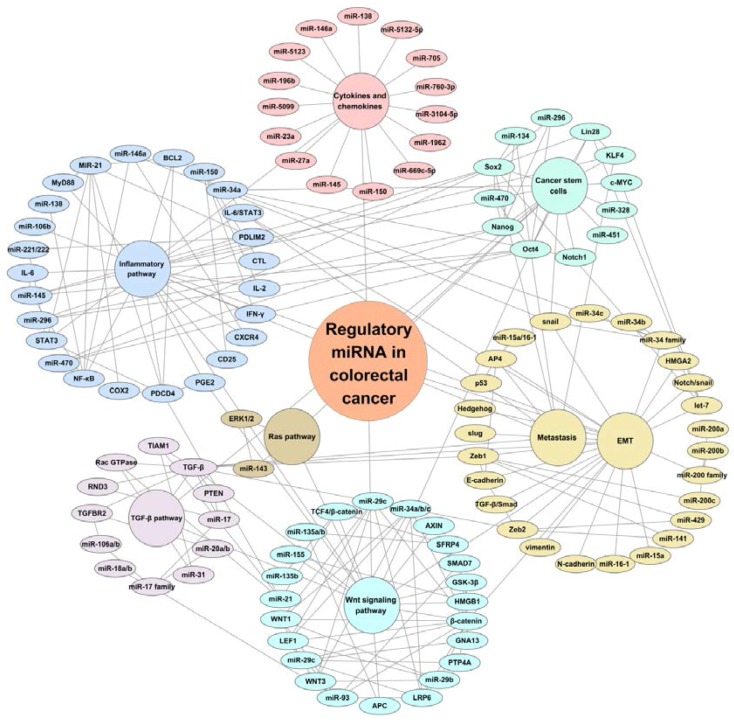
Scheme of the regulatory miRNAs and signaling pathways in colorectal cancer. There is a regulatory network linked to miRNAs and their targets, such as miRNAs regulating Wnt, Ras, transforming growth factor β (TGF-β),inflammatory, cytokine and chenmokines, cancer stem cells, epithelial-mesenchymal (EMT), and metastasis signaling pathways (in different colors). In addition, these signaling pathways are linked and regulated by each other.

Colorectal cancers are mainly caused by the activation of driven genes in the oncogenic signaling pathways, such as Wnt, Ras, TGF-β, and inflammatory signaling pathways, and these signaling pathways are regulated by individual miRNA or a clusters/groups of miRNAs (Figure 1).

### 3.1. miRNAs and Wnt Signaling

The deregulation of Wnt signaling is one of the most frequently-changed events in colorectal cancer, resulting from *APC* (adenomatosis polyposis coli) gene mutations that have been observed in about 75% of sporadic CRC [30,31,32]. *APC*, *AXIN*, and *GSK-3β* form a destruction complex, and this complex is essential for the phosphorylation and degradation of β-catenin. *APC* mutation and Wnt ligands present could cause the loss of the destroying function and cytoplastic accumulation of β-catenin, a key effector of Wnt signaling, resulting in β-catenin nuclear translocation and interacting with *TCF4* (transcription factor 4), and activating the transcription of target genes, such as cyclin D1 and c-myc.

Recent studies have revealed that miRNAs can also regulate the Wnt signaling pathway by targeting the key elements of the Wnt pathway. For example, miR-135a/b is overexpressed in CRC and is able to directly target *APC*, leading to the repression of APC expression and to the upregulation of Wnt signaling [33]. miR-135a/b is also predicted to target and inhibit secreted frizzled-related protein 4 (SFRP4), the latter is a Wnt/β-catenin inhibitor through binding and repressing extracellular Wnt proteins [34].On the other hand, miR-135b can be transcriptionally activated by β-catenin/TCF4, which shows that miR-135b is significantly upregulated in human CRC and in the tumors of an Apc mutation mouse model [35]. Unlike miR-135, miR-21 can increase β-catenin nuclear translocation and promote tumorigenesis in colorectal cancer [36]. Vice versa, miRNA-21 can also target the β-catenin signaling pathway and enhance Wnt-driven epithelial carcinogenesis [37]. Like miR-21, miR-155 is also a Wnt/β-catenin stimulator through targeting the Wnt signaling inhibitor HMGB1 and indirectly increasing Wnt/β-catenin expression [38,39].

Another group of miRNAs have tumor suppressive properties and can directly target and repress Wnt signaling. For example, the miR-34 family (miR-34a/b/c) can directly target Wnt ligands *WNT1, WNT3*, and *LRP6*, and *β-catenin* and *LEF1*, transcription factors that interact with β-catenin [40]. In addition, miR-29b, miR-29c, and miR-93 can inhibit Wnt ligands and β-catenin-mediated functions, e.g., miR-29c targets *GNA13* and *PTP4A* that are negative regulators of GSK3β, a kinase that phosphorylates β-catenin and triggers its degradation; miR-93 targets *SMAD7*, which promotes nuclear accumulation of β-catenin [41,42].

### 3.2. miRNAs and RAS Pathway

The *RAS* gene family is well characterized and plays important roles in regulating cell proliferation, apoptosis, differentiation, and migration and, therefore, acts as an oncogene [43]. There are three human isoforms, *NRAS*, *HRAS*, and *KRAS*, and *KRAS* is frequently mutated in colorectal cancers with 30%–40% mutation rates, moreover, *KRAS* mutations have been shown to be well-associated with poorer outcomes, in terms of shorter survival times, and being more aggressive and drug-resistant [44,45,46].

Since the *RAS* genes have several miRNA let-7 binding sites at the 3′-UTR, let-7 targets and regulates *KRAS* gene expression, and the reduction of let-7 in cancer tissues is correlated with higher KRAS mRNA expression, suggesting the regulatory roles of let-7 miRNAs in KRAS [47,48,49]. Indeed, the in vitro study showed that let-7 miRNA suppressed colon cancer growth and proliferation [50], in contrast, transfection with let-7a precursor miRNA significantly inhibited cancer cell growth and reduced the expression of KRAS and c-MYC [50].

miR-143 has been shown downregulation in colorectal cancer tissues to bind to the 3′-UTR of the *KRAS* gene [28,51], and reduced expression of miR-143 led to cell proliferation *in vitro*, which is linked to the increased expression of *KRAS* [51], thus, miR-143, like let-7, acts as tumor suppressor in *KRAS*-driven colorectal carcinogenesis [29], whereas treating colorectal cancer cells with a miR-143 mimic or overexpressing miR-143 resulted in cell proliferation and downregulation of KRAS and ERK1/2 [51]. Unlike let-7 and miR-143, miR-31 has been shown to negatively regulate KRAS inhibitor RASA1; thus, miR-31 could be a potent enhancer of *KRAS* in colorectal cancer [52,53,54].

### 3.3. miRNAs and TGF-β Pathway

Transforming growth factor-β (TGF-β) is a multitasking cytokine and TGF-β signaling pathway plays important physical and pathological roles in regulating cell proliferation, differentiation, apoptosis, migration, invasion, and modification of the microenvironment and cancer metastasis [55,56,57]. TGF-β binds two distinct receptor serine/threonine kinases, the type I receptors (TβRI) and type II receptors (TβRII), and activates SMADs and non-SMADs signaling pathways [56,57,58]. Interestingly, the TGF-β signaling pathway has a paradoxical effect on cancer biology: it maintains proliferation and differentiation in normal cells and early-stage cancer cells, but promotes cancer cell invasion and metastasis in late-stage cancers [59]. The TGF-β signaling pathway can regulate, and be regulated by, a series of molecular and signaling pathways, where miRNAs have been shown to play important roles, and among them, the miR-17 family seems to have crosstalk with the TGF-β signaling pathway [60,61].

The miR-17 family has eight miRNAs, including miR-17, miR-18a/b, miR-20a/b, miR-93, and miR-106a/b, and three of them (i.e., miR-17, miR-18a, and miR-20a) are transcribed from the miR-17-92 locus.MiR-17 targets and inhibits *PTEN* [62] and *RHOE* (*RND3*) [63], a tumor suppressor that is downregulated in CRC and exhibits inhibition of cancer cell invasion [64].

Several lines of evidence have also shown that miR-20a, another member of the miR-17 family, promotes cancer progression by facilitating CRC cell line migration and invasion and upregulating the expression of epithelial-mesenchymal transition (EMT) markers, neutralizes the growth-repressive properties of TGF-β, and further enhances the ability of TGF-β to drive cancer cell migration, invasion, and metastasis [55,65,66]. Like miR-20a, miR-106a/b seems also to enhance EMT and metastasis by targeting TGF-β receptor*TGFBR2*. miR-106a is highly expressed in metastatic CRC cell lines, and promotes cancer cell migration and invasion, but miR-106b has been reported to exert stimulatory and inhibitory effects on the migration and EMT of CRC cell lines [67,68,69]. Considering theabove, the studies to date have suggested that the miR-17 family promotes CRC metastasis through interaction with TGF-β signaling, as well as other pathways that modulate EMT.

### 3.4. miRNAs and Inflammatory Pathway

Epidemiology and experimental studies have strongly suggested that inflammatory signaling pathways are also key drivers of CRC [5,70,71,72], and the upregulation of chemokines and cytokinesis the major characteristic of inflammation-associated colorectal cancer [73,74]. The studies from us and others have demonstrated that the increase of cytokines (e.g., COX2, NF-κB, TNFα, IL-1β, IL6, etc.), C-X-C Motif Chemokine Ligand (CXCL) family members (CXCL1, CXCL2, CXCL6, CXCL8, and CXCL12), and CXC receptors (CXCRs) could be the cause of the malignant transformation of chronic colitis, resulting from gut microbiota disorder and mucosa barrier deficiency, leading to genetic and epigenetic alterations and oncogenic signaling activation (e.g., Wnt, Ras, PI3K/AKT/STAT3, etc.) [5,16,75,76], whereas the inflammatory signaling pathway could be regulated by miRNAs. 

COX2 is a prostaglandin-endoperoxide synthase and is responsible for generating PGE2, a pro-inflammatory prostaglandin that also activates the Wnt signaling pathway and is frequently overexpressed in CRC [77,78]. More studies have demonstrated that programmed cell death 4 (PDCD4) is reduced during CRC tumorigenesis, along with the transformation of normal tissue to adenocarcinoma COX2/PGE2-mediated repression of PDCD4 occurring via the induction of miR-21 [79], and the reduction of PDCD4 is well-associated with a shorter survival time of CRC patients [80]. miR-21 is one of the most prominent oncogenic miRNAs in colorectal cancer and has pro-tumorigenic properties in many other cancers [81].

miR-21 is also involved in the regulation of NF-κB and MyD88, an adapter of Toll-like receptors (TLRs) needed for NF-κB activation by TLR ligands [82,83]. In the azoxymethane (AOM)/DSS mouse model of CRC, genetic inactivation of miR-21 reduced the tumor burden and decreased the expression of pro-inflammatory cytokines, and the loss of miR-21 in tumors also increased PDCD4 expression and apoptosis, but reduced the expression of activated STAT3 and BCL2 [81]. Similar tomiR-21, miR-221 and miR-222 can also positively activate NF-κB and STAT3 by indirectly modulating their protein stability through miR-221/222-mediatedpositive feedback loops to increase expression levels of RelA and STAT3 [84].Therefore, the miR-21 family acts as a key modulator in oncogenic and inflammatory signaling pathways in which the miR-21 family maintains a positive loop with the modulation factors PDCD4, NF-κB, and STAT3.

miR-34a is a regulator of IL-6/STAT3 signaling in colorectal cancer. Rokavec et al. treated human colorectal cancer cells with cytokine IL-6and found that IL-6 activated the oncogenic STAT3 transcription factor, which directly represses miR-34a via a conserved STAT3-binding site in the first intron [85]. Repression of miR-34a was required for IL-6-induced EMT and invasion. An active IL-6R/STAT3/miR-34aloop was necessary for EMT, invasion, and metastasis of CRC cell lines and was associated with nodal and distant metastasis in CRC patients [85].

Our recent studies have also revealed tumor-suppressive functions of miRNAs (miR-138, miR-145, miR-146a, and miR-150) that were significantly reduced in colitis and colitis-associated CRC [16]. As to their regulatory functions, several studies have demonstrated that miR-138 activates the central cytokine NF-κB, promotes lipid raft formation in esophageal squamous cell carcinoma [86], and induction of miR-138 by pro-inflammatory cytokines causes endothelial cell dysfunction [87]; miR-145 targets the SOX9/ADAM17 axis to regulate tumor-initiating cells’ properties in head and neck cancers and suppresses the IL-6-mediated paracrine signaling pathway in the tumor microenvironment [88]. Tumor necrosis factor-related apoptosis-inducing ligand (TRAIL) can induce miR-146a expression and the later suppresses CXCR4-mediated cancer cell migration [89]; and miR-150 interacts with cytokines and is downregulated by inflammation in cytotoxic T lymphocytes. Moreover, miR-150 acts with the miRNA network and controls perforin, eomesodermin, and IL-2Rα expression in differentiating CTLs and whose activity is modulated by IL-2, inflammation, and antigenic stimulation [90]. In addition, miR-150 plays a critical role in the development and function of invariant NKT cells via regulationof IFN-γ expression [91]. As reported by us [16], cytokines were significantly increased and miR-138, 145, 146a, and miR-150 were significantly decreased in *Muc2-/-* mouse colon and human colitis and colorectal cancer. Taken together, miR-138, 145, 146a, and miR-150 are well regulatorily interacted with cytokines and inflammatory factors in the development of chronic colitis and its malignant transformation.

## 4. Regulatory miRNAs in Cancer Stem Cells, Epithelial-Mesenchymal Transition (EMT), and Metastasis

### 4.1. miRNAs and Cancer Stem Cells

Emerging data have suggested that miRNAs have significant roles in regulating the function of normal cells and cancer stem cells (CSCs) through their interaction with various signaling pathways (Figure 1). The CSCs are a special population of cells that have two main properties of heterogeneity and plasticity [92], in terms of showing distinct characteristics compared to normal cancer cells, such as tumor maintenance, progression, invasion, recurrence, and chemo-resistance [92,93,94]. Therefore, CSCs are clinically important. 

Six major factors are required for stem cell pluripotency maintenance. They are Nanog, Sox2, Oct4, KLF4, Lin28, and c-Myc. In addition to the regulation of CSCs by Wnt, TGF-β, Ras, and inflammatory signaling pathways, the CSCs are also regulated by miRNAs [95,96,97]. It has been observed that several miRNAs, such as miR-470, miR-296, and miR-134 may inhibit the self-renewing factors Oct4, Sox2, and Nanog [98]. In contrast, miR-145 helps in cell differentiation through targeting of KLF4, Sox2, and Oct4 [99]. Moreover, Hwang et al. have reported that miRNA-146a regulates snail-dependent symmetric division of colorectal CSCs [100], and the snail-miR-146a-β-catenin loop plays significant roles in the symmetric division of colorectal CSCs. Xu et al. have reported that expression of miR-328 was reduced in colorectal CSCs, and that increased expression of miR-328 suppressed cancer cells invasiveness and sensitized chemotherapy [101]. Therefore, miR-328 might be a potential target for CRC therapy. Bitarte et al. have found that miR-451 suppressed the self-renewal, tumor malignancy, and recurrence of colorectal CSCs [102]. Bu et al. have reported that miR-34a, like miR-451, is also a tumor suppressor and is differentially expressed in differentiating and self-renewing colon progenitor cells [103]. Moreover, the expression levels of miR-34a can regulate differentiation and self-renewalin vitroand in vivo by targeting Notch signaling. In fact, numerous studies have demonstrated that the Notch signaling pathway is an important regulator in asymmetric division and plays essential roles in promoting self-renewal of gastrointestinal stem cells and in colonic cell lineage differentiation [13,104,105]. This is also supported by experimental studies showing that colorectal CSCs might share common properties with normal colon stem cells because the CSCs in colorectal cancer xenografts exhibit similar morphology heterogeneity and histopathology as the parental tumor [106,107].

### 4.2. miRNAs and EMT and Metastasis

Epithelial-mesenchymal transition (EMT) is a specific physiological and/or pathological event of transformation from epithelial cells to mesenchymal cells. Numerous studies have demonstrated that EMT is an early event of cancer metastasis [108,109], in which epithelial cells lose their polarities, intercellular junctions and epithelial-like characteristics, and acquire less-differentiation and spindle-like phenotypes. EMT includes cell morphology and genotype changes; for instance, the round and less-aggressive epithelial cells transform into spindle-like and more-aggressive cells, and the cells also encounter molecular reprogramming, including the loss of cell adhesion molecules, such as E-cadherin, and gain of the expression of vimentin, N-cadherin, snail, slug, and other interstitial or stromal proteins [109,110]. Thus, EMT cells exert higher capabilities of migration and invasion and, therefore, the EMT plays critical roles during the early stages of cancer invasion and metastasis.

It is well known that the EMT is regulated by oncogenic signaling pathways, including Wnt/β-catenin, Notch/snail, Hedgehog/MMPs, TGF-β/Smad, and EGFR/MAPK/JNK signaling pathways [109,110]. Recent findings have shown that miRNA expression is also important in regulating the EMT process, such as the miR-200 family, miR-34 family, let-7, and miR-15a/miR-16-1, etc., act via the EMT signaling pathway and enhances metastasis (Figure 1).

As described above, miRNAs regulate EMT in colorectal cancer, partly by regulating the expression of tumor suppressors and oncogenes, and partly by functioning as tumor suppressors or oncogenes themselves [14,111], promoting or repressing CRC, EMT, and metastasis. The miR-200 family, including miR-200a, miR-200b, miR-200c, miR-141, and miR-429, was found to target the complementary sites in the 3-UTR of Zeb1 and Zeb2 (repressors of E-cadherin and key regulators of EMT progression), resulting indirectly in the upregulated expression of E-cadherin. EMT activator TGF-β is produced by tumor cells and can trigger the expression of Zeb1/2. Interestingly, the expression of miR-141 and miR-200c could be suppressed by Zeb1; for instance, knockdown of Zeb1 leads to increases of miR-141, miR-200c, and E-cadherin expression, increases cell-cell adhesion, induces epithelial phenotype, and reduces cell migration and invasion [112].In contrast, overexpressing Zeb1 can facilitate EMT progression and promote cancer cell invasion via triggering a miRNA-mediated feed-forward loop. Vice versa, induction of miR-200 promotes the differentiation and inhibition of epithelial-mesenchymal-specific gene expression by downregulating the expression of Zeb1 and Zeb2. Thus, the ZEB/miR-200 feedback loop is the molecular motor of cellular plasticity in the development of, and in particular is a driving force for, cancer progression towards metastasis by controlling the state of cancer stem cells [113].

The miR-34 family (miR-34a, miR-34b, and miR-34c) exerts physiological functions involved in cell cycle progression, senescence, and apoptosis. The members target snail, one of the EMT-inducing transcription factors, and leads to the downregulation of Snail expression [114,115]. Furthermore, miR-34a can suppress tumor progression by inhibiting the IL-6R/STAT3/miR-34a feedback loop and by inhibiting IL-6-induced colorectal cancer cell EMT, invasiveness, and metastasis [85].

Transcription factor AP4 is a downstream target of p53. It can directly repress E-cadherin via a non-canonical AP4-binding motif and induces N-cadherin-mediated EMT in colorectal cancer. Recent studies have shown that miR-15a/16-1 targets AP4 3'-UTR, attenuates EMT progression, and metastasis [116,117]. Recent findings have shown that miRNA let-7 is associated with cancer EMT and metastasis by targeting HMGA2 [118], and that let-7 could be repressed by Lin28, in conjunction with *OCT4*, *SOX2*, and *KLF4*, to promote colorectal cancer progression and metastasis [119]. Moreover, the TGF-β signaling pathway promotes cancer cell migration and invasion, which could be enhanced by miR-21 and miR-31 via suppressing TIAM1, a guanidine exchange factor of Rac GTPase and a direct target of both miR-21 and miR-31 [120].

## 5. Conclusions and Perspectives

As summarized in this review and in Figure 1, miRNAs represent a novel category of critical regulators in modulating tumor suppressors and oncogenes, or acting as tumor suppressors or oncogenes themselves, in colorectal cancer, participating in the regulation of colorectal cancer initiation, progression, stemness, EMT, metastasis, and chemotherapy response, they also represent promising biomarkers for CRC diagnosis and therapeutic targets for precision oncology. Better understanding of the regulatory roles of miRNAs in colorectal cancer initiation and progression may provide new insights of developing mini-invasive diagnostic tools for CRC screening and personalized therapy. Despite the numerous studies of miRNAs and extensive analyses of their expression, the roles and functions of many individual miRNAs in CRC remain poorly understood. Therefore, the integrated analysis of multiple miRNA targets for a given miRNA, and the integrated bioinformatic analysis of mRNAs, proteins, copy number variants, and mutations from the available public online databases (e.g., The Cancer Genome Atlas database and Oncomine database), are strongly needed. Improving our understanding of the mechanisms of regulatory interactions of mRNAs with the signaling pathways in colorectal cancer stem cells will aid in determining the genes responsible for progression, metastasis, and recurrence and, finally, in developing personalized prevention and therapy.

## Abbreviations

miRNA	microRNA
CRC	colorectal cancer
CSC	cancer stem cells
EMT	epithelial-mesenchymal transition
